# Computational enhancer prediction: evaluation and improvements

**DOI:** 10.1186/s12859-019-2781-x

**Published:** 2019-04-05

**Authors:** Hasiba Asma, Marc S. Halfon

**Affiliations:** 1 Program in Genetics, Genomics, and Bioinformatics, University at Buffalo-State University of New York, 701 Ellicott St, Buffalo, NY 14203 USA; 2 Department of Biochemistry, University at Buffalo-State University of New York, 701 Ellicott St, Buffalo, NY 14203 USA; 3 Department of Biological Sciences, University at Buffalo-State University of New York, 701 Ellicott St, Buffalo, NY 14203 USA; 4 Department of Biomedical Informatics, University at Buffalo-State University of New York, 701 Ellicott St, Buffalo, NY 14203 USA; 5 NY State Center of Excellence in Bioinformatics and Life Sciences, 701 Ellicott St, Buffalo, NY 14203 USA; 6 Molecular and Cellular Biology Department and Program in Cancer Genetics, Roswell Park Comprehensive Cancer Center, Buffalo, NY 14263 USA

## Abstract

**Background:**

Identifying transcriptional enhancers and other *cis*-regulatory modules (CRMs) is an important goal of post-sequencing genome annotation. Computational approaches provide a useful complement to empirical methods for CRM discovery, but it is critical that we develop effective means to evaluate their performance in terms of estimating their sensitivity and specificity.

**Results:**

We introduce here *pCRMeval*, a pipeline for in silico evaluation of any enhancer prediction tools that are flexible enough to be applied to the *Drosophila melanogaster* genome. *pCRMeval* compares the result of predictions with the extensive existing knowledge of experimentally-validated *Drosophila* CRMs in order to estimate the precision and relative sensitivity of the prediction method. In the case of supervised prediction methods—when training data composed of validated CRMs are used—*pCRMeval* can also assess the sensitivity of specific training sets. We demonstrate the utility of *pCRMeval* through evaluation of our SCRMshaw CRM prediction method and training data. By measuring the impact of different parameters on SCRMshaw performance, as assessed by *pCRMeval,* we develop a more robust version of SCRMshaw, SCRMshaw_HD, that improves the number of predictions while maintaining sensitivity and specificity. Our analysis also demonstrates that SCRMshaw_HD, when applied to increasingly less well-assembled genomes, maintains its strong predictive power with only a minor drop-off in performance.

**Conclusion:**

Our *pCRMeval* pipeline provides a general framework for evaluation that can be applied to any CRM prediction method, particularly a supervised method. While we make use of it here primarily to test and improve a particular method for CRM prediction, SCRMshaw, *pCRMeval* should provide a valuable platform to the research community not only for evaluating individual methods, but also for comparing between competing methods.

**Electronic supplementary material:**

The online version of this article (10.1186/s12859-019-2781-x) contains supplementary material, which is available to authorized users.

## Background

Transcriptional enhancers, or more broadly, *cis*-regulatory modules (CRMs), are essential building blocks of gene regulatory networks [1, 2]. Present upstream, downstream, and within introns of their associated genes, and often at a considerable genomic distance, CRM sequences serve as scaffolds for the binding of transcription factors and chromatin modifying enzymes. Their identification is critical for understanding the spatial and temporal regulation of metazoan gene expression.

As part of the contemporary arsenal of methods for CRM discovery, computational approaches have proven to be an important complement to experimental ones [3, 4]. Computational CRM discovery has several advantages, including low cost, rapid results, and no requirement for access to cell lines, antibodies, tissue samples, and other expensive and/or limiting biological resources and assays. This is of particular benefit when working with non-model organisms, for which there may be genome sequence but frequently not extensive other genomic data. However, the existence of multiple computational CRM discovery methods leads to a familiar problem: with many software approaches, how do how do we know which ones perform the best? Given time and resource constraints, typically only a limited number of predicted regulatory elements from a given method can be validated empirically, and a comprehensive set of CRM and non-CRM sequences does not exist for any metazoan genome. A systematic evaluation of a dozen in silico CRM discovery methods was carried out in 2010 [5], but a similar assessment of the many approaches developed since that time has not been performed.

To help address this, we have developed *pCRMeval*, a pipeline for in silico evaluation of predicted CRMs with particular application to supervised methods trained on known CRMs. Our pipeline provides a general framework that can be used to evaluate any CRM prediction tool flexible enough to be applied to the *Drosophila melanogaster* genome. We leverage the REDfly database of experimentally-validated *Drosophila* CRMs ([6]; over 23,900 as of 1 Jan 2019) to compare CRM prediction results and to make estimates of the sensitivity and specificity of a given method, or of specific training data applied to that method. Because REDfly includes information about the spatio-temporal specificity of CRMs, our evaluation platform allows for an assessment of not just a method’s CRM-discovery ability, but also for its ability to predict CRMs with a specific activity profile.

We demonstrate here the usefulness of the *pCRMeval* pipeline by using it to evaluate parameters and training sets for our own SCRMshaw prediction method [7–9]. We find that SCRMshaw results vary depending on the chromosomal position at which analysis begins, and develop a refinement to the SCRMshaw protocol that provides robustness to starting parameters. This updated protocol predicts (on average) a greater number of CRMs while maintaining similar sensitivity and precision as the original, less robust method. We also test the impact of degree of genome assembly on SCRMshaw’s performance and find that SCRMshaw remains efficacious for CRM discovery even when assemblies are poor.

## Results

### The *pCRMeval* evaluation pipeline

We developed a comprehensive pipeline, *pCRMeval*, for in silico evaluation of CRM prediction methods. We sought to keep the core requirements for the pipeline minimal in order to accommodate the widest range of different prediction approaches. Therefore, the only input absolutely required is a BED-formatted list of predicted CRMs. However, because our evaluation methods rely on the extensive set of experimentally validated CRMs available for *Drosophila melanogaster*, the predictions to be evaluated must be based on the *Drosophila* genome. Optionally, a list of CRMs used as training data, which will be a component of most supervised machine learning approaches, can also be provided; this allows for evaluation of the performance of specific training sets in addition to evaluation of the method as a whole.

### Performance measures

Assessing the performance of CRM prediction approaches is challenging, as the true full set of CRMs in the genome, and knowledge of each CRM’s complete activity profile, is not known. As a result, accurate calculations of sensitivity and precision of CRM prediction is not possible. In light of this, we devised several performance measures to evaluate different aspects of CRM prediction, as follows (see Methods for details):

- REDfly recovery

The most straightforward way to assess the overall performance of a CRM prediction method is to compare the prediction results with the set of true CRMs. While the full set is not known, almost 24,000 experimentally-validated *Drosophila* enhancers are contained in the REDfly database [6]. We measure *REDfly recovery* by determining what fraction of predicted CRMs overlap the known sequences in REDfly.

REDfly, while extensive, does not contain a complete catalog of all true CRMs in the *Drosophila* genome. As a result, *REDfly recovery* can be interpreted in two ways. A low recovery could indicate that the majority of predictions correspond to previously undiscovered CRMs, suggesting exceptionally good prediction performance. However, with close to 24,000 CRMs currently in REDfly, we expect that at least some number of predictions will correspond to known CRMs. We therefore generally place more weight on the second alternative, that lower *REDfly recovery* correlates with decreased performance and a higher false-positive prediction rate, as fewer true CRMs are being discovered. In a supervised (trained) setting, interpretation of *REDfly recovery* is influenced by the number of known CRMs matching the expression characteristics of each training set. The former scenario increases in likelihood when the number of non-training REDfly CRMs annotated with the training set expression pattern is a very small fraction of the total REDfly CRMs, as there is thus a lower expectation that appropriate known CRMs will be identified. Conversely, the latter interpretation gains strength with increased numbers of appropriately-annotated CRMs. Additional file 1: Table S1 provides these numbers for the various training sets discussed in this study. Despite its ambiguity in interpretation, we find *REDfly recovery* to be a useful metric when used in conjunction with the other performance measures. It is particularly helpful in comparing different prediction methods head-to-head using the same set of training data. Moreover, as REDfly continues to grow, this measure will gain in utility.

- Training set sensitivity

A method trained on a set of known CRMs should be able to predict all members of that same set. For optional use with supervised methods, therefore, we calculate *training set sensitivity* as the fraction of training CRMs successfully recovered.

- Expression pattern precision

REDfly annotates CRMs with the developmental stages and tissues where they are active. These data can be used to assess the precision of CRM prediction in cases where a method is trained to recover CRMs with a particular activity profile. *Expression pattern precision* measures the fraction of predicted CRMs driving the expected expression pattern out of the total number of predicted CRMs driving any annotated expression pattern.

- Additional measures

Along with *REDfly recovery, training set sensitivity,* and *expression pattern precision, pCRMeval* calculates two additional sensitivity measures: *REDfly recall* and *expression pattern recall* (see Methods). At present, we have not found these measures useful in our CRM prediction method evaluations, and do not discuss them further here. However, we include them in the software pipeline in the event that they prove helpful under a different evaluation framework, or with different input data.

### Output

*pCRMeval* generates a tab-delimited summary table amenable for downstream analysis. An R markdown script [10] is also provided for presenting the data in graphical form.

### Application of *pCRMeval* to SCRMshaw

We previously developed an effective method for computational CRM discovery, SCRMshaw (for Supervised *cis*-Regulatory Module discovery) [7–9]. SCRMshaw uses a training set composed of known CRMs defined by a common functional characterization (e.g., “nervous system,” “midgut”) to build a statistical model that captures their short DNA subsequence (*k*-mer) count distribution and compares it to that of a set of non-CRM “background” sequences in a machine-learning framework. The trained model is then used to score overlapping sequence windows in the genome, and the highest-scoring windows are predicted to be CRMs. In empirical validation experiments using reporter genes in transgenic animals, success rates have averaged roughly 80%, and when trained on *Drosophila melanogaster* sequences, SCRMshaw is even able to discover CRMs in a cross-species fashion in genomes as far diverged as the 345 Ma honeybee (*Apis mellifera*) genome with a ~ 75% true-positive rate [11, 12].

Our empirical testing suggested that some training sets are more effective than others at high-quality prediction, but the large number of possible training sets makes it infeasible to attempt empirical validation of each set. Similarly, it is not practical to attempt to use in vivo validation to assess the effect of changing various SCRMshaw parameters in a systematic way. We therefore applied *pCRMeval* to SCRMshaw for purposes of evaluating different training sets and SCRMshaw parameters.

### Training set evaluation

**Fig. 1:**
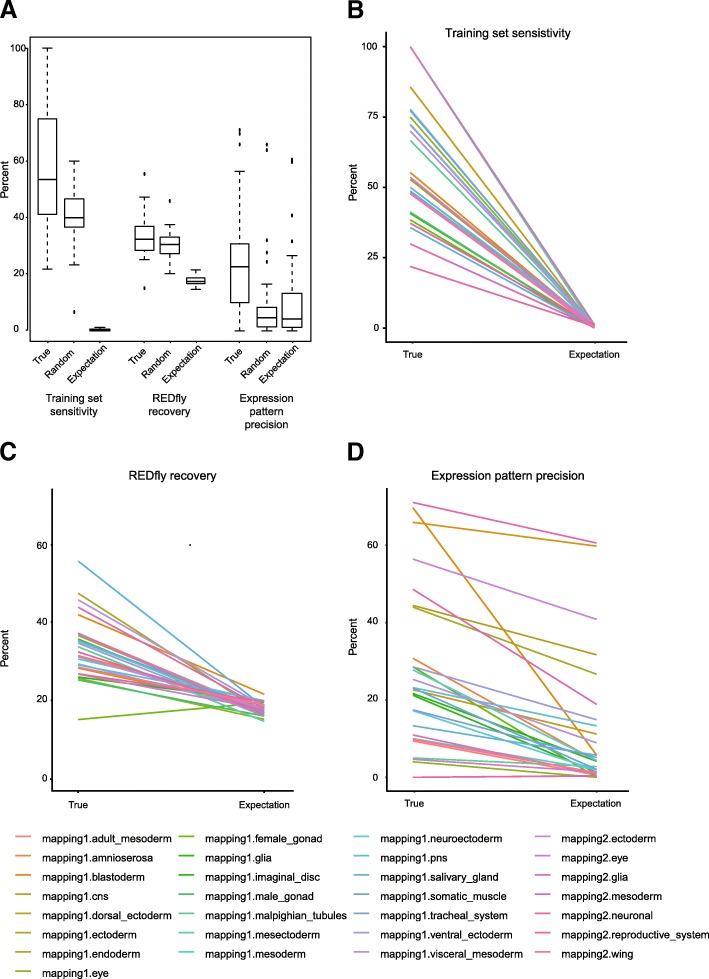
Performance evaluation for SCRMshaw using *pCRMeval*. pCRMeval demonstrates that SCRMshaw, when training on real CRM data, performs better than either random training data or random expectation. **a** Aggregate performance for *training set sensitivity*, *REDfly recovery,* and *expression pattern precision* for 29 true training sets, 62 random training sets, and random expectation. **b-d** Comparison of *training set sensitivity* (**b**), *REDfly recovery* (**c**), and *expression pattern precision* (**d**) for true predictions versus random expectation for each of the 29 training sets

We used the *pCRMeval* pipeline to assess 29 different training sets used with SCRMshaw, following the SCRMshaw-HD protocol (an improved variant of the default SCRMshaw protocol, described below) (Fig. 1, Additional file 2: Table S2).

Results are summarized in Fig. 1a. *REDfly recovery* ranged from 15 to 55% (median 32%), versus a random expectation of 17%. The median *training set sensitivity* was 54% (range 21–100%) versus a random expectation of 0.47%. *Expression pattern precision* ranged from 0 to 71% (median 23%, expectation 4%). One-to-one comparisons for individual training sets with their respective random expectations are shown in Fig. 1b-d and Additional file 2: Table S2.

We also compared the performance of each of our training sets to the averaged results from running SCRMshaw on 62 “random” training sets composed of 30 randomly-selected regions from the *Drosophila* non-coding genome (Fig. 1a, “random”). SCRMshaw consistently showed higher performance using real training data as compared to the random training sets (random training set values: *training set sensitivity* 41%, range 6–60%; *REDfly recovery* 28%, range 20–45%; *expression pattern precision* 4.6%, range 0–65%).

**Fig. 2:**
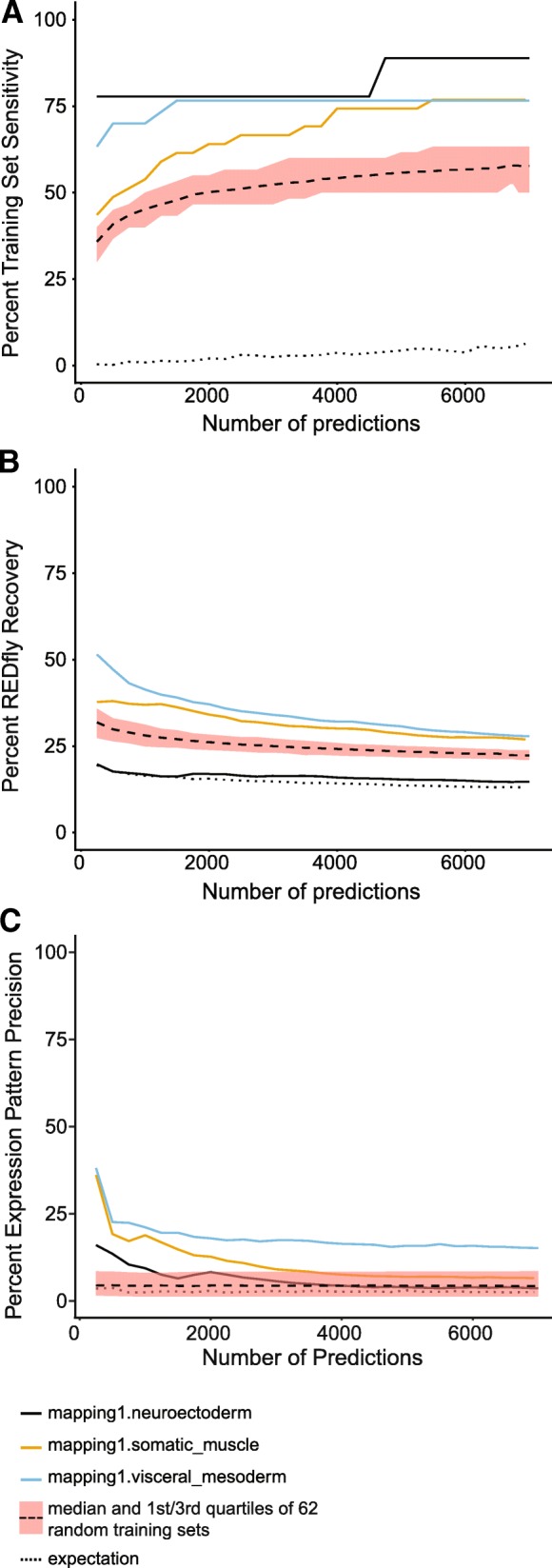
Performance evaluation for SCRMshaw using *pCRMeval* on a continuous scale. (**a**) *Training set sensitivity,* (**b**) *REDfly recovery,* and (**c**) e*xpression pattern precision* for selected training sets (solid lines) compared to the median percentage (dashed line) and 1st and 3rd quartiles (shaded region) of 62 random training sets, and to random expectation (dotted line). Black solid line, mapping 1.neuroectoderm; orange solid line, mapping1.somatic_muscle; blue solid line, mapping1.visceral_mesoderm

In addition, we evaluated the performance of each individual training set in a semi-continuous fashion by calculating *training set sensitivity*, *REDfly recovery*, and *expression pattern precision* every 250 predictions for the top 7000 predictions (Fig. 2a-c, Additional file 3: Figure S1 and Additional file 4: Table S3). *Training set sensitivity* is always substantially better across the entire range of predictions compared to both random training sets (black dashed line with shaded area) and to random expectation (black dotted line) (Fig. 2a). In contrast, while *REDfly recovery* is always at least moderately better than random expectation, some training sets perform less well than the random training sets (e.g. mapping1.neuroectoderm; Fig. 2b). For *expression pattern precision*, almost all of the training sets perform better than both random expectation and random training sets for their top few hundred to thousand predictions, but performance declines markedly with larger numbers of predictions (Fig. 2c). Both *REDfly recovery* and *expression pattern precision* decrease as the number of predictions increases, suggesting increasing numbers of false-positive predictions. In general, we find our cutoffs for top predictions (see Methods) to be a reasonable tradeoff between number of predictions and number of false positives.

Based on its performance for each of our three measures, we categorized each training set as “good,” “intermediate,” or “poor” (Table 1). Good training sets perform well by all three measures, with the training set outperforming random training sets by at least 10% for *training set sensitivity,* at least 5% for *expression pattern precision,* and at least 4% for *REDfly recovery*. Intermediate training sets perform well compared to random training sets in at least two of the three measures using a slightly more generous difference of at least 7% for *training set sensitivity,* 5% for *expression pattern precision*, and 3% for *REDfly recovery.* Poor training sets perform worse than or equivalent to random training sets for at least two of the three measures. Out of our 29 training sets, ten meet the criteria for being a “good” set and eleven fall into the intermediate category. The remaining eight sets are classified as poor. Note, however, that even the sets in the “poor” category perform better than random in at least one of the three measures.

**Table 1:** Evaluation of training sets based on their performance of measures

Class	Training set Name	Difference to random^a^ in REDfly recovery at cutoff	Difference to random in training set sensitivity at cutoff	Difference to random in expression pattern precision at cutoff
Good	mapping1.blastoderm	11.28777	15.29412	65.08609
	mapping1.ectoderm	6.000862	12.83019	39.83357
	mapping1.endoderm	16.77476	10	39.38912
	mapping1.glia	5.071332	60	16.60124
	mapping1.tracheal_system	6.446144	37.27273	12.83098
	mapping1.visceral_mesoderm	15.07699	30	20.70558
	mapping2.glia	6.586659	60	6.326622
	mapping1.adult_mesoderm	6.49964	26.66667	4.764122
	mapping1.mesectoderm	4.541295	60	5.389122
	mapping1.ventral_ectoderm	4.046977	32.22222	23.96055
Intermediate	mapping1.amnioserosa	0.839151	35	26.15835
	mapping1.malpighian_tubules	3.107168	26.66667	0.389122
	mapping1.dorsal_ectoderm	1.833739	45.71429	18.03063
	mapping1.mesoderm	−0.00957	32.41379	23.1669
	mapping1.neuroectoderm	−1.26327	37.77778	12.6305
	mapping1.pns	−1.52394	10	18.57753
	mapping1.salivary_gland	24.95711	−4.28571	8.722456
	mapping1.somatic_muscle	4.778675	8.717949	18.46605
	mapping2.ectoderm	0.548001	8	51.79938
	mapping2.mesoderm	13.1938	−10.0935	44.00023
	mapping2.wing	−2.08398	7.727273	5.225188
Poor	mapping1.cns	−2.33838	0.909091	61.32319
	mapping1.eye	−4.91517	35	−0.61088
	mapping2.eye	−3.869981	13.57143	0.040285
	mapping1.female_gonad	−15.4483	−1.53846	23.96055
	mapping1.imaginal_disc	−4.60542	0.740741	17.05579
	mapping1.male_gonad	−5.34252	1.176471	−4.61088
	mapping2.neuronal	−3.78756	−2.7451	66.44175
	mapping2.reproductive_system	1.796743	−18.125	−4.61088

^a^ “Random” refers to the averaged results of 62 random training sets of 30 sequences each (see text)

### Application to a second CRM prediction method

As an illustration of the utility of *pCRMeval* for evaluating other CRM prediction methods, we assessed the recent CRM predictions by [13]. We obtained numbers for *training set sensitivity* similar to those that were reported (94%, compared to the reported 98%). Interestingly, we observed a considerably higher *REDfly recovery*. Whereas Arbel et al. [13] report that 364 of their predicted CRMs overlap known REDfly CRMs and 822 are completely novel (31% *REDfly recovery* value), *pCRMeval* yields a *REDfly recovery* of 72% (859 known CRMs, 327 novel). Some of this discrepancy may be due to differences in the stringency used to call a sequence a known CRM. For *pCRMeval* the minimum overlap was set to only 10% (corresponding to a minimum of 100 bp overlap). Increasing that stringency to 50% (500 bp), for instance, would yield a more similar *REDfly recovery* value of 413. Because they focused on pregastrula gene regulation in *Drosophila*, which falls into the REDfly category “blastoderm,” we were able to assess *expression pattern precision* as well, for a value of 40%. These results from *pCRMeval* support the contention of Arbel et al. [13] that they developed an accurate and sensitive predictor of early *Drosophila* CRMs.

### SCRMshaw is sensitive to the position of the first analysis window

**Fig. 3:**
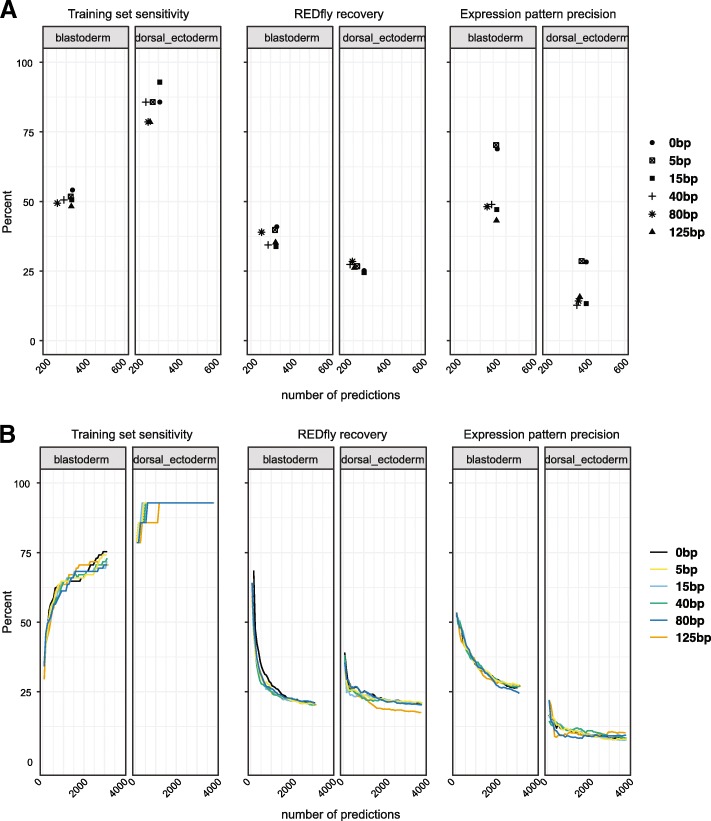
SCRMshaw results vary based on analysis starting position. Results are shown based on *pCRMeval* assessment of *training set sensitivity*, *REDfly recovery,* and *expression pattern precision* for two representative trainings sets (“mapping1.blastoderm,” “mapping1.dorsal_ectoderm”) with starting position offsets of 0, 5, 15, 40, 80 and 125 base pairs. **a** Results using a fixed cutoff. **b** Results using a continuous scale

We next used *pCRMeval* to assess various SCRMshaw parameters. By default, SCRMshaw evaluates 500 bp windows with a 250 bp overlap, starting from the first base of each chromosome (or assembled scaffold). We were curious to determine whether the starting position of these windows on the chromosome affects the output, that is, how sensitive SCRMshaw is to the specific windows that are analyzed. To test this, we ran SCRMshaw on the *Drosophila* genome using the default parameters, but instead of starting at the beginning of each chromosome took windows starting from the 5th, 15th, 40th, 80th, and 125th base pairs. We analyzed each run using *pCRMeval* and assessed each of the three evaluation measures at both a fixed cutoff and in a semi-continuous fashion, as above. To our surprise, we observed a small but definite variation in performance for each training set due to differences in chromosome starting position (Fig. 3, Table 2, Additional file 5: Figure S2, and Additional file 6: Table S4).

**Table 2:** Performance comparison of default SCRMshaw with different starting points with SCRMshaw-HD

	Number of top predictions		Training set sensitivity (%)		REDfly recovery (%)		Expression pattern sensitivity	
	5 to 125 bp offsets (range)	SCRMshaw-HD	5 to 125 bp offsets (range)	SCRMshaw-HD	5 to 125 bp offsets (range)	SCRMshaw-HD	5 to 125 bp offsets (range)	SCRMshaw-HD
mapping1.blastoderm	254–329	328	48.24–51.76	**55.29**	33.84–38.98	**41.84**	43.15–48.90	**69.70**
mapping1.dorsal_ectoderm	234–306	**330**	78.57–92.86	85.71	24.51–28.46	**32.39**	12.75–15.70	**22.64**
mapping1.mesoderm	241–278	**332**	58.62–72.41	**72.41**	22.27–25.74	**30.55**	19.92–27.01	**27.78**
mapping2.wing	364–550	453	38.64–54.55	47.73	22.86–24.45	**28.47**	4.31–7.43	**9.84**

Bolded values are greater than or equal to the maximum value observed for default SCRMshaw

With a fixed cutoff, calculated for each SCRMshaw run based on the elbow point of the score curve (see Methods), each starting point gave a slightly different number of top predictions (Fig. 3, x-axis), with moderate variation in *REDfly recovery*, *training set sensitivity*, and *expression pattern precision*. For example, for the training set “mapping1.blastoderm,” the *REDfly recovery* ranges from 34 to 40%, *training set sensitivity* from 48 to 54% and *expression pattern precision* from 43 to 70% (Fig. 3). On the continuous scale (i.e., calculated at each 250 predictions until 7000) we similarly observed differences based on each starting point (Fig. 3, Additional file 7: Figure S3, and Additional file 8: Table S5).

### A more robust approach: SCRMshaw-HD

**Fig. 4:**
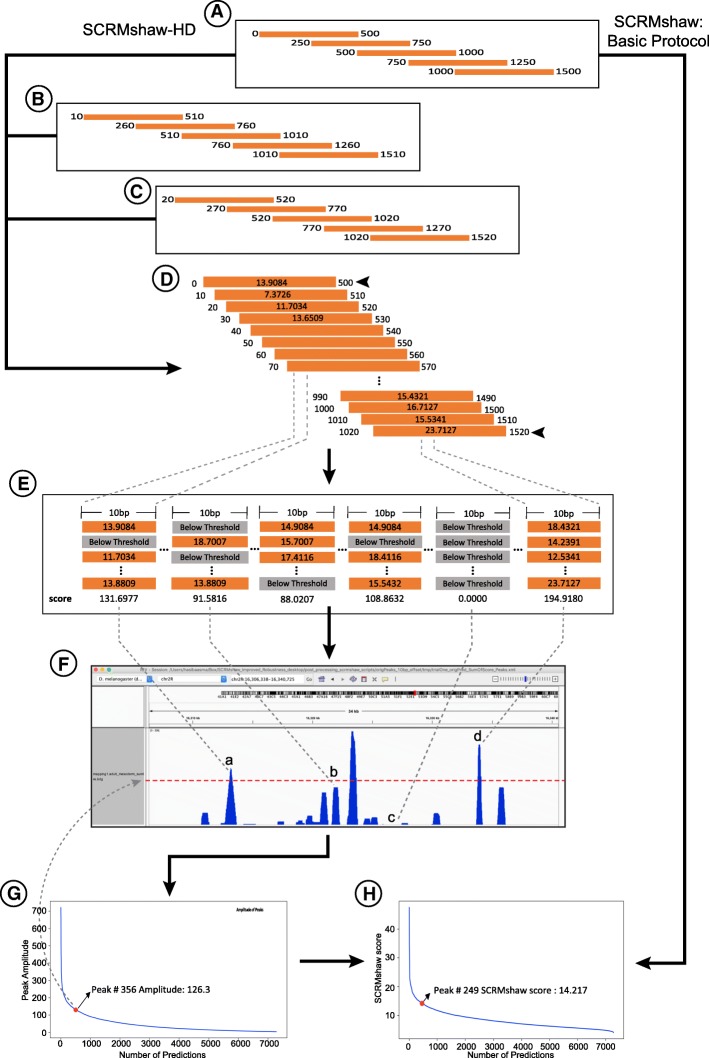
The SCRMshaw-HD protocol. The new, more robust SCRMshaw-HD protocol is shown to the left, with the default SCRMshaw protocol to the right. **a** In both protocols, 500 bp windows are scored with a 250 bp offset between windows. **b**, **c** For SCRMshaw-HD, this process is parallelized by running 25 instances of SCRMshaw simultaneously, with each instance starting its first window at a different starting points corresponding to 0, 10, 20, 30, …, 240 bp from each chromosome/scaffold end. **d** The output from the individual SCRMshaw runs is concatenated into a single output representing 500 bp windows with 10 bp offsets across the entire genome. **e** The SCRMshaw scores for each 10 bp genomic window are summed, with any individual score (orange boxes) below the value of the 5000th ranked score reassigned to zero (gray boxes). The summed scores (**f**) are used as the basis for peak calling. Any peaks with amplitude above the selected amplitude threshold (**g**, red dot) are then evaluated for SCRMshaw score (**h**, red dot; see Methods for details). Peaks meeting both criteria (e.g., peak “d” in panel **f**) are accepted as “top predictions.” Peaks that either fall below the amplitude cutoff (e.g. “b” in panel **f**), or which pass the amplitude cutoff but not the score cutoff (“a” in panel **f**) are not considered top predictions. In default SCRMshaw (right side of figure), those predictions with SCRMshaw score above the elbow point of the curve of all ranked SCRMshaw scores are considered to be “top” predictions (**h**, red dot)

Although the differences are relatively minor, these results indicate that the default 250-bp window shift size used by SCRMshaw is sensitive to where the windows begin. We reasoned that a smaller shift would fix this problem by scoring a more comprehensive set of sequence windows, but would have significantly increased computational cost. We devised a new variant of the SCRMshaw protocol, “SCRMshaw-HD,” to balance the need for more tightly overlapping windows with the computational demands (Fig. 4). To obtain higher resolution scoring profiles, we run SCRMshaw using a wide range of starting positions, from 0 to 240 bp, with a step size of 10 bp (i.e., 0, 10, 20, 30,…,240). This is achieved by use of a new “offset” parameter in the SCRMshaw software, while maintaining the default 500 bp window with 250 bp overlap. We chose 10 bp as the step size because the performance difference with 5 or 15 bp offsets as compared to the original (0 bp) starting position tends to be negligible (Fig. 3, Additional file 5: Figure S2, Additional file 7: Figure S3 and Table 2). Each of the 25 starting positions can be run as a separate instance of SCRMshaw, allowing for simple parallelization on a computing cluster (Fig. 4a-c). Note that this is equivalent to simply changing the default shift size parameter from 250 bp to 10 bp and following the basic SCRMshaw protocol (e.g., as described in [9]), which allows for execution on single processor. However, the latter approach would significantly boost the execution time, particularly for a large genome.

A post-processing script concatenates the output from each of the 25 individual instances (Fig. 4d). We then sum the SCRMshaw scores from each prediction overlapping every 10 bp window in the genome, resetting scores below a cutoff threshold (defined as the SCRMshaw score at the 5000th ranked prediction) to zero (Fig. 4e). The resulting set of windows with their summed SCRMshaw score is used as the input to a peak-calling algorithm (Fig. 4f), with the peaks passing a cutoff criterion considered as “top” predictions (see Methods for details; Fig. 4g, h).

We used our evaluation pipeline to compare the results from SCRMshaw-HD to those from the SCRMshaw runs for individual starting positions. For almost all of the training sets, performance of SCRMshaw-HD for *training set sensitivity* fell within or exceeded the range seen with default SCRMshaw, while SCRMshaw-HD performance exceeded that of the default method for both *REDfly recovery* and *expression pattern precision* (Table 2). Interestingly, the number of top predictions also tended to be larger for SCRMshaw-HD (Table 2). SCRMshaw-HD is therefore not only more robust to chromosome starting positions, but generally provides a larger number of predicted CRMs while maintaining or improving multiple measures of sensitivity and specificity.

### Effect of degree of genome assembly

Because many newly-sequenced species do not yet have full chromosome-level assemblies, we were interested to know how SCRMshaw would perform on less-well-assembled genomes. The improved robustness conferred by the SCRMshaw-HD approach might be particularly valuable for this, as such genomes have many individual scaffolds and therefore more potential starting positions for window generation. To explore this, we simulated a range of poorly-assembled genomes by “fragmenting” the *Drosophila* genome into a series of shorter sequences, and comparing CRM predictions run on the fragmented genomes to predictions using the fully assembled genome.

**Fig. 5:**
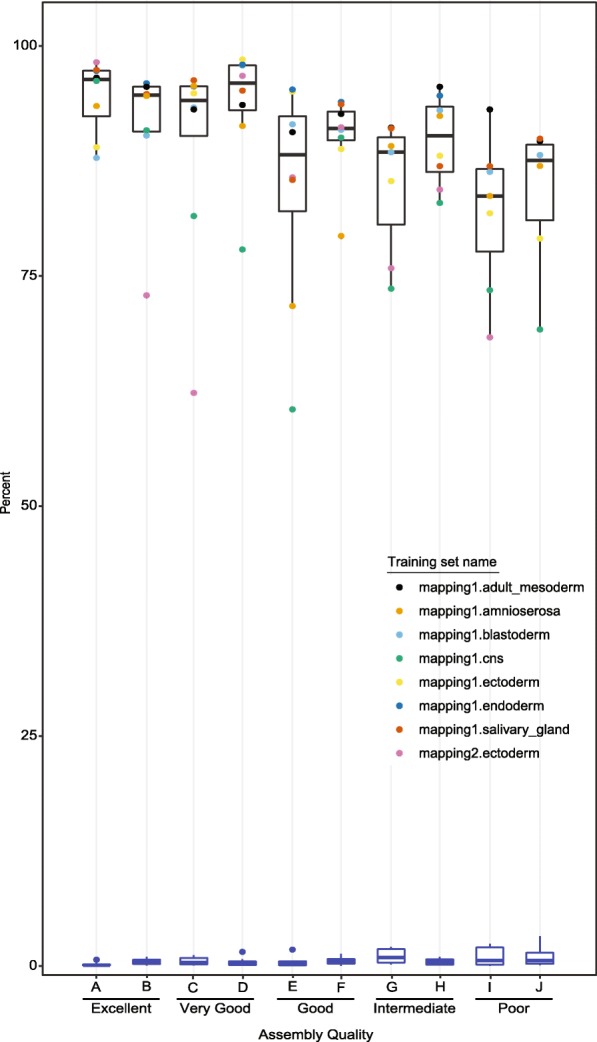
Degree of genome assembly has a minimal impact on SCRMshaw performance. Black boxplots (top) show the aggregate percentage of true positives for eight representative training sets (each shown as different colored point), and blue boxplots (bottom) the aggregate percentage of false positives for the same sets, over a range of simulated qualities of genome assembly. For details about genomes “A” through “J” see Table 3

**Fig. 6:**
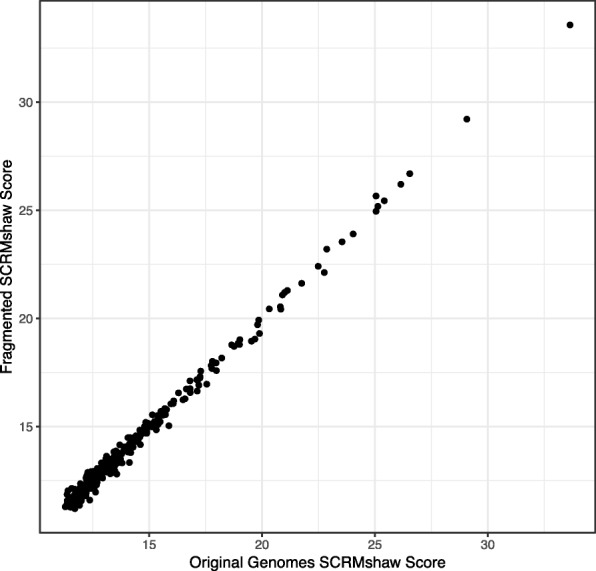
Correlation between SCRMshaw scores of corresponding windows in the native and simulated genomes

To make the simulations realistic, we mimicked the scaffold-length distribution of real insect species with varying quality of genome assembly, ranging from “excellent” to “poor” (see Methods). We then used SCRMshaw-HD to obtain the top CRM predictions from each simulated genome, and from the native *Drosophila* genome. For purposes of evaluation, we considered all CRM predictions for the native genome above our score cutoff to be true positives, and everything below the cutoff to be true negatives. For the simulated genomes, the percent true positives (sensitivity) could then be calculated as the number of predictions above the cutoff that mapped to true positives in native genome. Overall, we observed a minimal decline in true positives—less than 15% on average—in moving from the best to the worst-assembled genomes, and only a negligible increase in false positive rate (~ 1%; Fig. 5). Although a few training sets (e.g., mapping1.cns, mapping2.ectoderm) showed high variability in true positive rate, fluctuating by as much as 40% (Fig. 5), these changes were not correlated with the extent of genome assembly and are likely more indicative of training set quality than genome quality. As an additional assessment, we compared the SCRMshaw scores of corresponding windows in the simulated vs. native genomes, and consistently observed a high correlation between the two (r > 0.99; Fig. 6). From these two measures, we conclude that SCRMshaw remains effective for CRM prediction even in cases where the genome assembly is poor.

## Discussion

### A more robust SCRMshaw approach

In this study, we have developed an improved version of our SCRMshaw method, SCRMshaw-HD, in which we predict enhancers using a higher density of genome coverage coupled with a peak-calling algorithm to select the top CRM predictions. These modifications make SCRMshaw more robust to initial placement of the scoring windows on the chromosome while simultaneously providing a slightly larger number of predictions, without decreasing sensitivity and specificity. The increased robustness is likely to be of particular importance when analyzing less-completely-assembled genomes, which have increased numbers of “starting” positions when the number of unassembled scaffolds is high. Our analysis demonstrates that the new SCRMshaw-HD method is successful in predicting CRMs effectively even from relatively less-assembled genomes, with only a minor drop-off in performance. This is a welcome finding, as most newly-sequenced genomes do not have complete assemblies.

However, there is a computational cost to the improved SCRMshaw-HD method, as the number of sequence windows to be examined increases 25-fold. While we provide a simple parallelization solution for those with access to a computing cluster, execution times will be significantly longer if SCRMshaw-HD is run using a single processor. Because the degree of difference between the results from the original SCRMshaw method and SCRMshaw-HD is modest, using SCRMshaw in its original form remains an acceptable alternative for those without access to a cluster.

### SCRMshaw training set assessment

Although we have demonstrated its effectiveness in several prior studies [7, 8, 11, 12, 14], *pCRMeval* allowed us for the first time to conduct an unbiased assessment of SCRMshaw on a training set by training set basis. Our results confirm SCRMshaw’s basic utility as a CRM-discovery method—virtually every tested training set performed better than random expectation, for all measures—but also provide useful insights both into aspects in need of improvement, and into CRM biology in general. For instance, median *training set sensitivity* was only 54%, suggesting that many training sets contain sequences that are not good fits to the predominant CRM sequence model for their set. This has been noted previously [7] and is not surprising given that these training sets were compiled using fairly low-granularity expression pattern data. Using *pCRMeval* to test performance, we will better be able to compile better, more cohesive training sets that should improve predictive performance. Preliminary testing with a few hand-picked, carefully assembled training sets has confirmed this both in silico and in in vivo validation experiments ([15] and unpublished results). Interestingly, *training set sensitivity* and *REDfly recovery* using training sets consisting of random genomic sequences consistently scored better than random expectation. Given that the *Drosophila* genome is compact, with a high proportion of non-repeat non-coding sequence likely devoted to gene regulation, this suggests that SCRMshaw is adept at discerning “generic” regulatory signatures even in the absence of a strong regulatory model dominating the training set. This helps explain why in empirical evaluation studies SCRMshaw has consistently performed better at discovering CRMs than in discovering CRMs that exclusively match the training set expression pattern [7, 8], and may also contribute to SCRMshaw’s demonstrated success in cross-species CRM discovery [11, 12, 14]. These generic signatures most likely consist of binding sequences for common transcription factor families, such as E-boxes and homeodomain binding sites, which can be bound by many different transcription factors and are known to be present in a large fraction of CRMs. A number of our current training sets performed only equivalent to (or worse) than the random training sets on one or more measures. These sets may lack a strong common regulatory signature and are in need of reconstitution with a more well-defined set of similarly-functioning CRMs. In a similar vein, we note that we find *expression pattern recall*—the sensitivity of predicting all known CRMs with expression characteristics matching those of the training set—to be quite low (median 4%, range 0–14%; Additional file 2: Table S2). We ascribe this to the fact that the expression pattern categories we use are broad and likely contain numerous CRMs that are not responsive to the regulatory signature defined by the training set. More fine-grained annotation and compilation of expression pattern categories should help to alleviate this problem and lead to improved expression pattern recall.

### *pCRMeval* vs. in vivo validation

Our empirical validation results have frequently demonstrated better performance than what is inferred by the *pCRMeval* results. For instance, in vivo validation of predictions from the “mapping1.blastoderm” training set yielded a 100% true positive rate/40% pattern precision rate (*n* = 5, [7]), while predictions from “mapping1.mesoderm” and “mapping1.somatic_muscle” had an 83% true positive rate/60% pattern precision rate (*n* = 12, [8]). This is compared to *pCRMeval* values of 42%/70% and ~ 33%/26% *REDfly recovery*/*expression pattern precision* for “mapping1.mesoderm” and “mapping1.somatic_muscle,” respectively. The “mapping2.wing” training set enabled cross-species predictions in the beetle *Tribolium castaneum* with a greater than 90% true positive rate as assessed by overlap with FAIRE-predicted CRMs [14], despite this training set being only an “intermediate” performer by *pCRMeval* (28%/10%)*.* Similarly, while the *pCRMeval* value of 40% for *expression pattern precision* that we determined for the method described by Arbel et al. [13] is toward the high end of the range we saw with our SCRMshaw datasets—consistent with the highly selected training data used—it is considerably below the 91% in vivo validation rate that study obtained. We ascribe these discrepancies to the fact that REDfly only contains known CRMs, and that expression pattern annotations are often incomplete as they are based only on features chosen to be described by the authors of the papers REDfly curates. Thus, it is important that *pCRMeval* results not be considered as an exact reflection of performance. Nevertheless, *pCRMeval* serves as a useful means for comparisons between training sets, prediction methods, parameter choices, and the like, and can provide an estimated lower bound for prediction sensitivity and precision.

### Comparing CRM prediction methods

Although our *pCRMeval* pipeline was designed with SCRMshaw in mind, it is easily applied to other CRM prediction methods, in particular supervised (trained) methods. Indeed, one interest of ours was to compare the performance of a number of current approaches, along the lines of the important but becoming outdated assessment previously performed by Su et al. [5]. Unfortunately, we found that we were unable to apply a sufficient number of the methods we selected due to myriad issues including inaccessibility via the published URLs, missing and/or obsolete dependencies, or failure to obtain a successful software installation despite reasonable efforts to modify or update the code. This finding is in line with a recent study that found over 77% of computational biology software tools either “difficult to install” or unable to be installed altogether [16]. Nevertheless, as demonstrated by our analysis of the Arbel et al. [13] prediction data discussed above, our evaluation pipeline is suitable for use with any working CRM prediction method that can be applied to the *Drosophila* genome and produces a list of predicted CRM coordinates as output.

## Conclusions

The *pCRMeval* pipeline allows for assessment of CRM-discovery methods in terms of their sensitivity and precision with respect to a large collection of known CRMs. When applied to our proven SCRMshaw method, it enabled construction of an improved, more robust protocol, characterization of multiple sets of training data, and an examination of the effects of genome assembly on CRM discovery. *pCRMeval* thus provides both a convenient platform for comparing the relative performance of CRM-discovery methods and a useful means for optimizing individual methods, and should be a useful addition to the CRM discovery software toolbox.

## Methods

### Datasets

The following files, based on *Drosophila* CRM data obtained from REDfly [6], were used for assessing *training set sensitivity*, *REDfly recovery*, and *expression pattern precision*:

#### REDfly CRMs file

CRMs from the REDfly database with length < 2 kb were downloaded in BED format (data downloaded July 14, 2017).

#### Expression-mapped CRMs file

This file contains the subset of REDfly CRMs that have associated tissue-pattern expression data, mapped to larger groupings as in [7] Table S6.

#### Training set sequences

Training data for SCRMshaw used the sets originally defined in [17]. An updated list of training data is available at https://github.com/HalfonLab/Training_sets.

### pCRMeval

*pCRMeval* is written in Python and requires the Python modules *pybedtools, statistics, scipy, numpy, pandas, csv* and *itertools*. The latest version can be obtained at https://github.com/HalfonLab/pCRMeval. *pCRMeval* calculates the following measures:

- REDfly recovery

*REDfly recovery* uses CRMs from the “REDfly CRMs” file. If training CRM information is available, the training CRMs are filtered out of this list. The remaining CRM sequences are sorted and merged using BEDTools *sort* and *merge* respectively [18]. BEDTools *intersect* is then used to determine the number of REDfly CRMs present in the predicted CRM set. For evaluations using SCRMshaw, the minimum overlap for *intersect* was set to 10% (−*f* 0.10), corresponding to a minimum of 50 bp overlap (as the shortest possible default SCRMshaw prediction is 500 bp). *REDfly recovery* is then calculated as the number of REDfly CRMs present in the predicted CRM set divided by the total number of predictions.

We also calculate *REDfly recall* by dividing the number of predictions found in REDfly by the total number of CRMs in REDfly. However, as REDfly is not a complete catalogue of all CRMs, and we expect that a good prediction method will identify both known and unknown CRMs, we do not find this to be a useful evaluation measure at this time.

- Training set sensitivity

*Training set sensitivity* is assessed by finding the overlap between training set CRMs and CRM predictions using BEDTools *intersect.* For evaluations using SCRMshaw, the minimum overlap for *intersect* was set to 10% (−*f* 0.10). *Training set sensitivity* is then calculated as the number of CRM predictions overlapping training set CRMs divided by the total number of CRMs present in the training set. Note that we do not calculate a training set precision value as there is little sense to such a measure: predicting CRMs not part of the training set is the goal of a supervised prediction method and such predictions are not false positives but rather, the desired outcomes.

- Expression pattern precision

To calculate *expression pattern precision*, BEDTools *intersect* is used to obtain overlaps between the expression-mapped CRMs file and the predicted CRMs (for evaluations using SCRMshaw, the minimum overlap for *intersect* was set to 10% (−*f* 0.10)). A simple count is then made of the number of CRMs with the expected expression pattern, divided by the total number of expression-mapped CRMs.

- Expression pattern recall

*Expression pattern recall* is calculated as the number of predictions with the correct expression pattern divided by the total number of CRMs known to drive expression similar to the training set.

### Permutation testing to calculate random expectation

To calculate the significance of each evaluation measure, the coordinates of the predicted CRMs are randomized using BEDTools *shuffle,* followed by calculation of each measure using the randomized coordinates instead of the actual CRM predictions. This is repeated 100 times to generate an empirical random distribution, and significance in determined by calculating a *z* score. The number of permutations can be adjusted using the *-s* argument on the *pCRMeval* command line. For evaluations using SCRMshaw, the *-excl* argument to BEDTools *shuffle* was used along with a file of exon coordinates, as SCRMshaw predictions were made using non-exon sequences only.

### Predictions using random training sets

Sixty two training sets containing 30 randomly-chosen non-coding sequences of varying length (median length, 745 bp, 1st/3rd quartile 360/1200 bp) were constructed using *randomWithSameGC.pl* (available as part of the SCRMshaw package). SCRMshaw was then trained on these training sets (random sequences) and the output evaluated using *pCRMeval* as described above.

### SCRMshaw output file

SCRMshaw was run on the *Drosophila melanogaster* release 6 genome with the following command line options:

- *perl /SCRMshaw/code/scrm.pl --genome TargetSequence --traindirlst TrainingSetList --gff annotationGffFile --thiwt 5000 --imm --hexmcd --pac --outdir --step 123 –lb*

For default SCRMshaw the parameter -*lb* is set to 0. For SCRMshaw-HD *-lb* is set to [0, 10, 20, 30,…,240] for parallel instances as described below (see SCRMshaw-HD).

Results are only reported here for the IMM component of the SCRMshaw score [8], but evaluations using the other scoring schemes were comparable (data not shown).

### Testing window starting positions

Offsets to the window starting positions were created by respectively deleting 0 bp, 5 bp, 15 bp, 40 bp, 80 bp, or 125 bp from the beginning of each of the primary chromosome arms and then using these trimmed genome sequences as input to SCRMshaw.

### SCRMshaw-HD

To generate higher-resolution scoring profiles, we introduced a command line option, the offset parameter -*lb,* to SCRMshaw. This offset value tells the algorithm to ignore any base pairs at the beginning of a chromosome/scaffold before this point, i.e., to start creating the analysis windows from this point in the genome. Offset values of 0 to 240 bp with a step size of 10 bp (i.e. 0, 10, 20, 30,…,240) were used for SCRMshaw-HD, with each individual offset being run as its own instance on a separate processor. This allows us to obtain the results for the multiple offsets within the same amount of time as required for default SCRMshaw.

### Postprocessing

The output from all instances were concatenated and passed to a post-processing Python script. The post-processing script first splits each 500-bp SCRMshaw prediction into individual 10-bp segments, with each segment retaining the score from the larger window. Note that with the exception of the ends of each chromosome/scaffold, there will be 25 individual 10-bp segments, each from a different SCRMshaw instance, for every 10-bp window in the genome. 10-bp segments sharing the same coordinates are merged and the sum of their SCRMshaw scores is calculated. Only the top 5000 SCRMshaw windows are scored; all other 500-bp windows are set to score = 0. The resulting file of 10-bp windows, with their summed scores, are then used as the input to MACs (Model-based Analysis of Chip-Seq; [19]) for peak calling. The MACs function *bdgpeakcall* is used for this purpose with default parameters and the score from the 5000th SCRMshaw prediction (i.e., the last non-zero SCRMshaw score used) as the value for parameter -*cutoff*.

### Cutoff script

For many applications, it will be useful to generate a list of top CRM predictions. To facilitate this task, we developed a simple python utility script, *“cutoff.py”,* which takes into account both the raw SCRMshaw scores and the summed scores (amplitude) of the SCRMshaw-HD peaks. The cutoff points are based on determining the “elbow” points of the score and amplitude curves (calculated as the point furthest from the line connecting the first and last points on the curve [20]) (Fig. 4g, h, red dots). For SCRMshaw-HD, defining the top predictions is a two-part process. First, all peaks with amplitude above the amplitude cutoff point are accepted (Fig. 4g). Then, the SCRMshaw score curve is constructed as follows: first, each peak is evaluated to determine the maximum SCRMshaw score for any sequence window within the peak (Fig. 4d, arrowheads). These scores are then ranked, and the “elbow” point calculated (Fig. 4h). Peaks which also pass this cutoff are accepted as the set of top predictions (which can then be passed to *pCRMeval* for evaluation; Fig. 4f, peak “d”).

For default SCRMshaw, a set of top predictions can be determined by simply finding the elbow point of the entire SCRMshaw score distribution and accepting all predictions with scores greater than or equal to the score at that point.

### Genome fragmentation

To test the effect of genome assembly, we downloaded the assembly statistics for a variety of arthropod species and categorized the quality of their genome assembly from “Excellent” (very well assembled) to “Poor” (very poorly assembled) (Table 3). Two species from each category were selected and the length of their sequenced scaffolds recorded. This length distribution was then mimicked for the *Drosophila melanogaster* genome by randomly picking segments with lengths drawn at random from each quartile of the distribution, starting from the beginning of each chromosome and proceeding iteratively until reaching the chromosome end. (The exception is for the “excellent” category, where one of the genomes was simulated simply by dividing each *D. melanogaster* chromosome arm into two halves.) This enabled us to achieve a simulated chromosome length distribution similar to that of the true genomes (Additional file 9: Figure S4). Each segment was then treated as a distinct chromosome/scaffold for the simulated genome. (For example, if original chrA has length of 1000 bp, and is broken into two fragments chrA:1–500 and chrA:501–1000, in the simulated genome these will be defined as two distinct chromosomes chrB:1–500 and chrC:1–500). Using the coordinates from the original genome, the sequence of each simulated chromosome was extracted using BEDTools *getfasta*. The simulated chromosomes were then annotated for coding/non-coding sequence by mapping the gene annotations from the original genome to the simulated genome.

**Table 3:** Categories and N50 distribution for genome assembly simulations

ID	Species	Scaffold N50	Category
A	*Drosophila melanogaster (divided chromosomes)*	NA	Excellent
B	*Aedes aegypti*	409,777,670	Excellent
C	*Ctenocephalides felis*	71,713,785	Very Good
D	*Apis mellifera*	13,619,445	Very Good
E	*Papilio xuthus*	6,198,915	Good
F	*Schizaphis graminum*	1,292,312	Good
G	*Pogonomyrmex barbatus*	819,605	Medium
H	*Bactrocera oleae*	139,566	Medium
I	*Lutzomyia longipalpis*	85,093	Poor
J	*Drosophila albomicans*	23,589	Poor

Simulated genomes were subject to CRM prediction using SCRMshaw-HD and the coordinates of the top predictions were then mapped back to their corresponding coordinates in the original genome. BEDTools *intersect* was used to calculate the number of predictions in common between the original and simulated genomes, with minimum overlap set at 50% (*−f* 0.50).

## Additional files

Additional file 1: **Table S1.** Sizes of expression-pattern annotated CRM groups in REDfly. (XLSX 13 kb)

Additional file 2: **Table S2.** Data for *pCRMeval* for 29 training sets at a fixed cutoff. Values for *training set sensitivity, REDfly recovery, REDfly recall, expression pattern precision* and *expression pattern recall* are reported. (XLSX 14 kb)

Additional file 3: **Figure S1.** Performance evaluation of SCRMshaw using *pCRMeval* on a semi-continuous scale. Performance of *training set sensitivity*, *REDfly recovery,* and *expression pattern precision* of 29 training sets. (PDF 23 kb)

Additional file 4: **Table S3.** Data for *pCRMeval* for 29 training sets on a semi-continuous scale. Values for *training set sensitivity, REDfly recovery, REDfly recall, expression pattern precision* and *expression pattern recall* are reported. (XLSX 276 kb)

Additional file 5: **Figure S2.** Results of SCRMshaw assessment by *pCRMeval* using a fixed cutoff. (i) Training set sensitivity, (ii) REDfly recovery, and (iii) expression pattern precision of 29 trainings sets with starting position offsets of 0, 5, 15, 40, 80 and 125 base pairs. (PDF 50 kb)

Additional file 6: **Table S4.** Data for *pCRMeval* for 29 training sets with varying offsets at a fixed cutoff. Values for *training set sensitivity, REDfly recovery, REDfly recall, expression pattern precision* and *expression pattern recall* with starting position offsets of 0, 5, 15, 40, 80 and 125 base pairs are reported. (XLSX 53 kb)

Additional file 7: **Figure S3.** Results of SCRMshaw assessment by *pCRMeval* on a semi-continuous scale. (i) Training set sensitivity, (ii) REDfly recovery, and (iii) expression pattern precision of 29 trainings sets with starting position offsets of 0, 5, 15, 40, 80 and 125 base pairs. (PDF 155 kb)

Additional file 8: **Table S5.** Data for *pCRMeval* for 29 training sets with varying offsets on a semi-continuous scale. Values for *training set sensitivity, REDfly recovery, REDfly recall, expression pattern precision* and *expression pattern recall* with starting position offsets of 0, 5, 15, 40, 80 and 125 base pairs are reported. (XLSX 2034 kb)

Additional file 9: **Figure S4.** Scaffold length distribution of real vs simulated genome. (PDF 494 kb)

## Abbreviation

CRM: *cis*-regulatory module
