# Supplementary figures and images for: Computational enhancer prediction: evaluation and improvements
Source: BMC Bioinformatics. 2019 Apr 5;20:174. doi: 10.1186/s12859-019-2781-x (PMC6451241; doi:10.1186/s12859-019-2781-x)

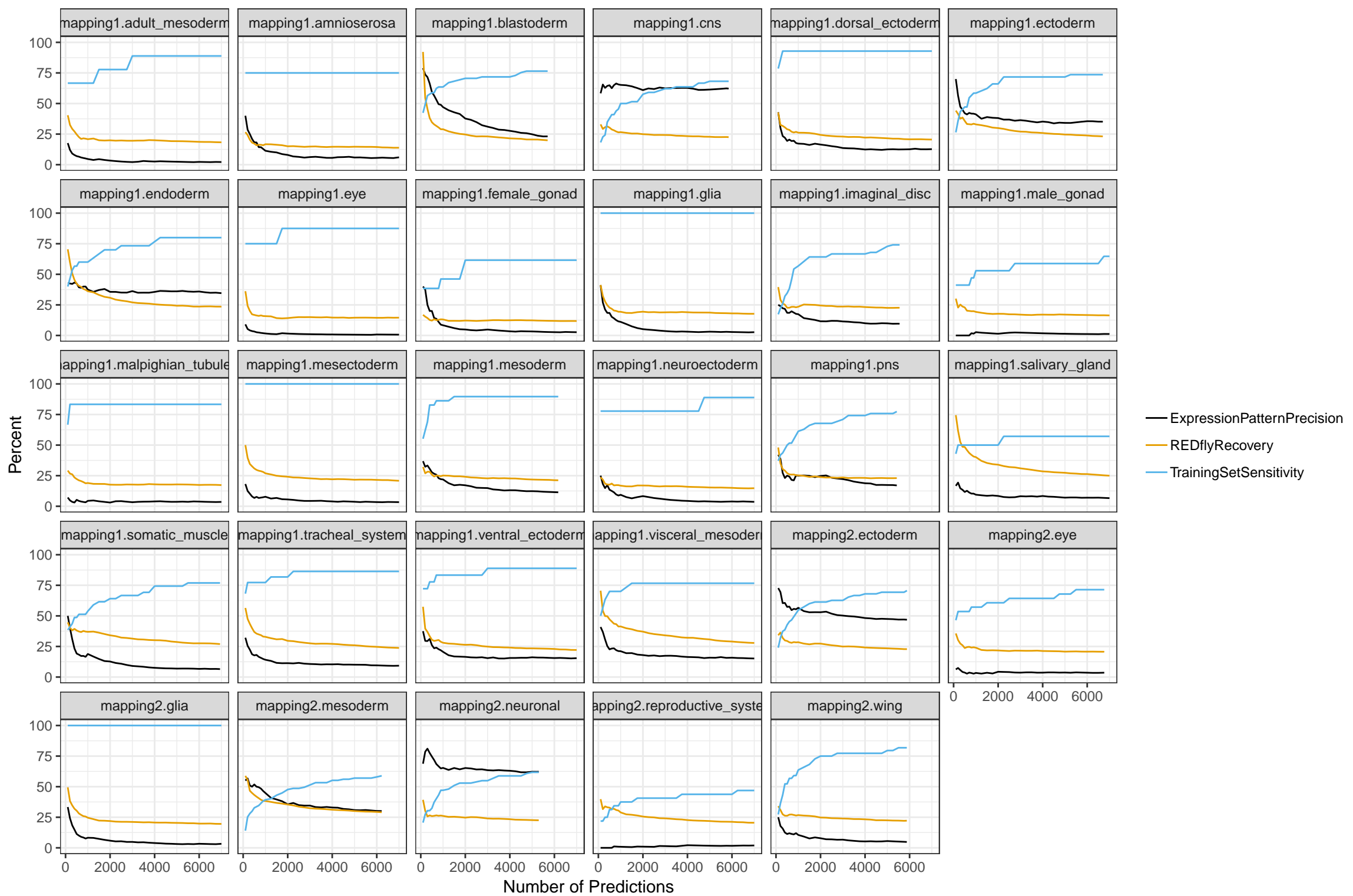

Supplement: Supplementary file 3 — Figure S1. Performance evaluation of SCRMshaw using pCRMeval on a semi-continuous scale. Performance of training set sensitivity, REDfly recovery, and expression pattern precision of 29 training sets. (PDF 23 kb) [file 12859_2019_2781_MOESM3_ESM.pdf]

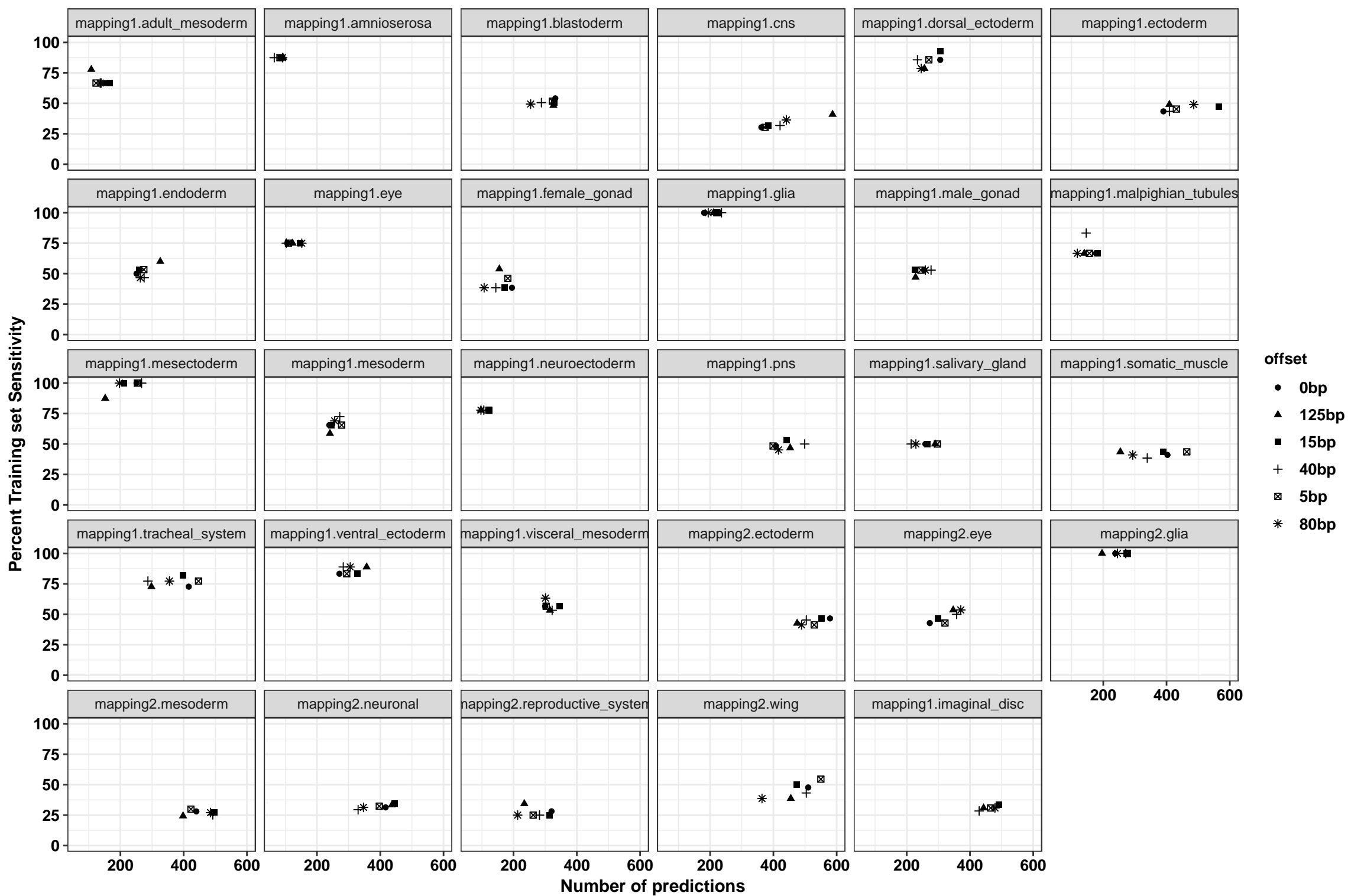

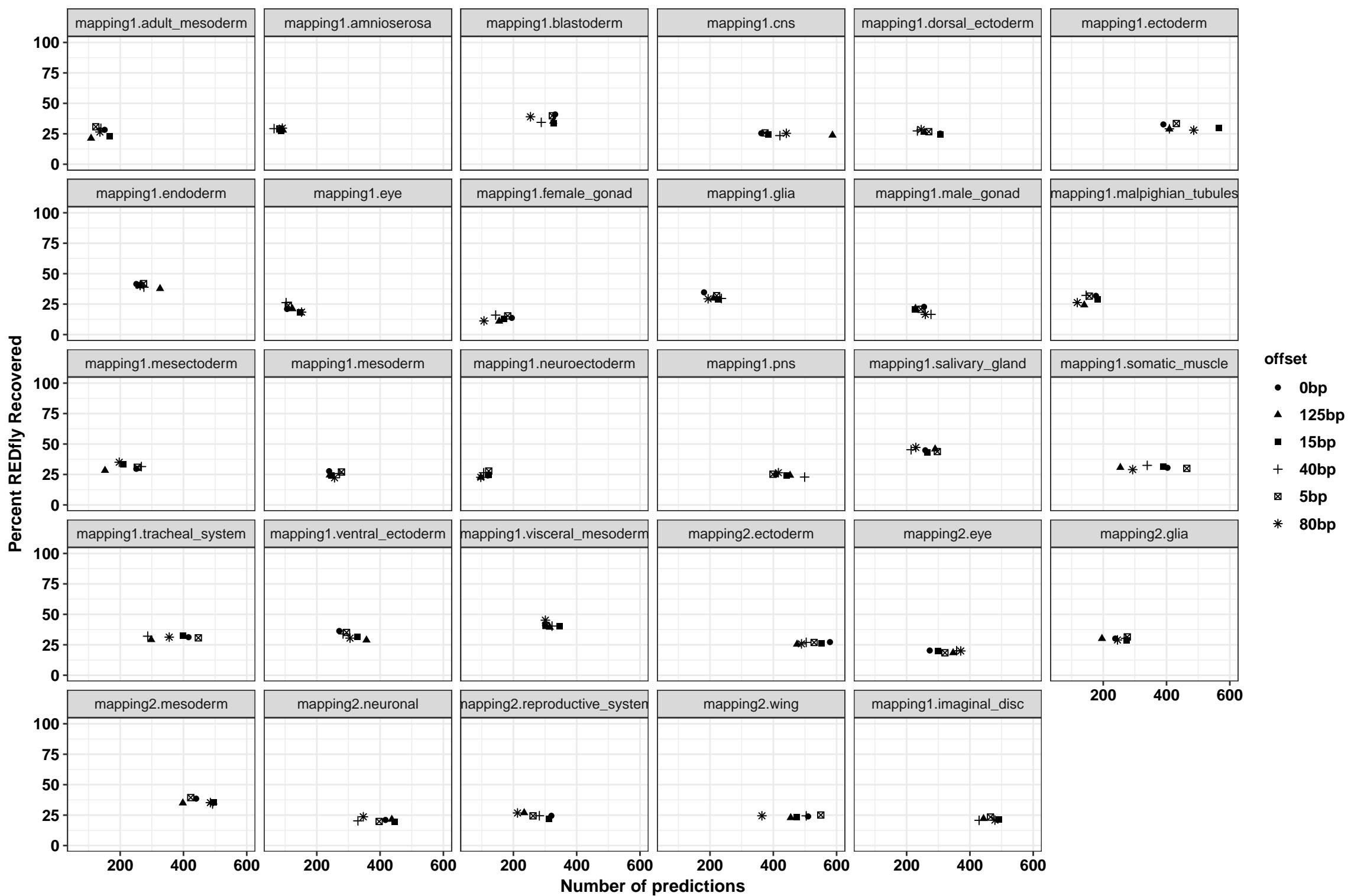

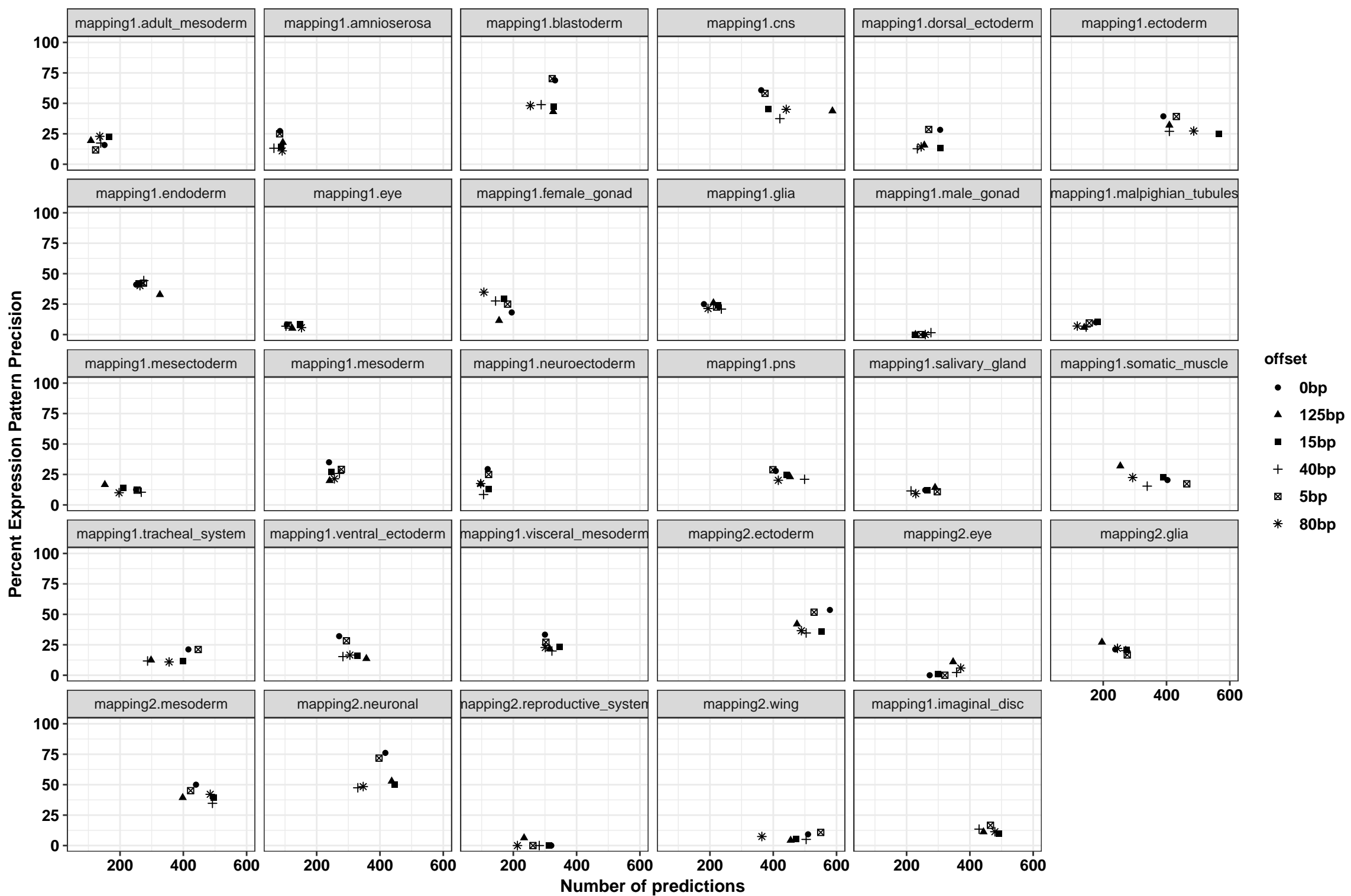

Supplement: Supplementary file 5 — Figure S2. Results of SCRMshaw assessment by pCRMeval using a fixed cutoff. (i) Training set sensitivity, (ii) REDfly recovery, and (iii) expression pattern precision of 29 trainings sets with starting position offsets of 0, 5, 15, 40, 80 and 125 base pairs. (PDF 50 kb) [file 12859_2019_2781_MOESM5_ESM.pdf]

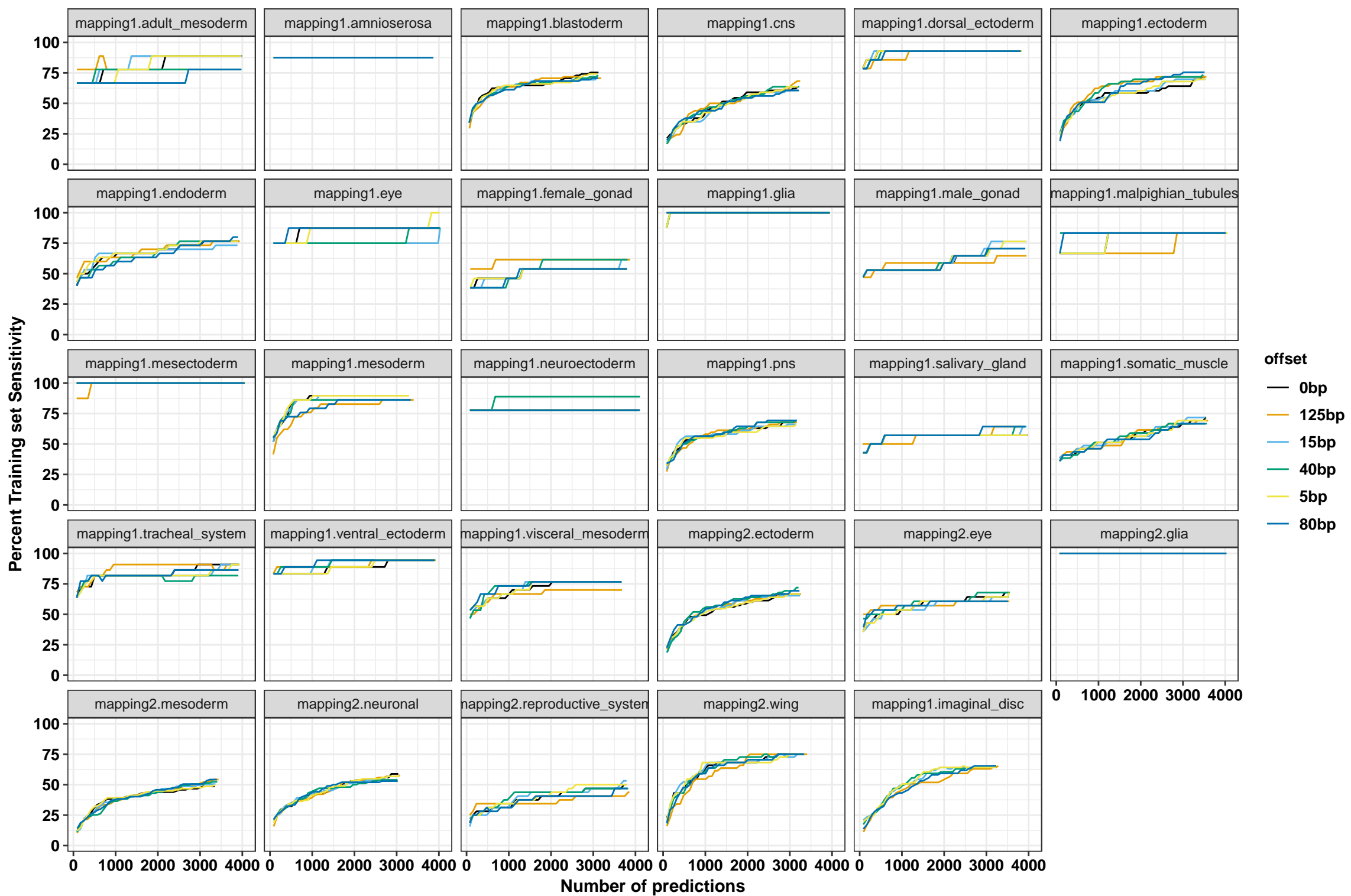

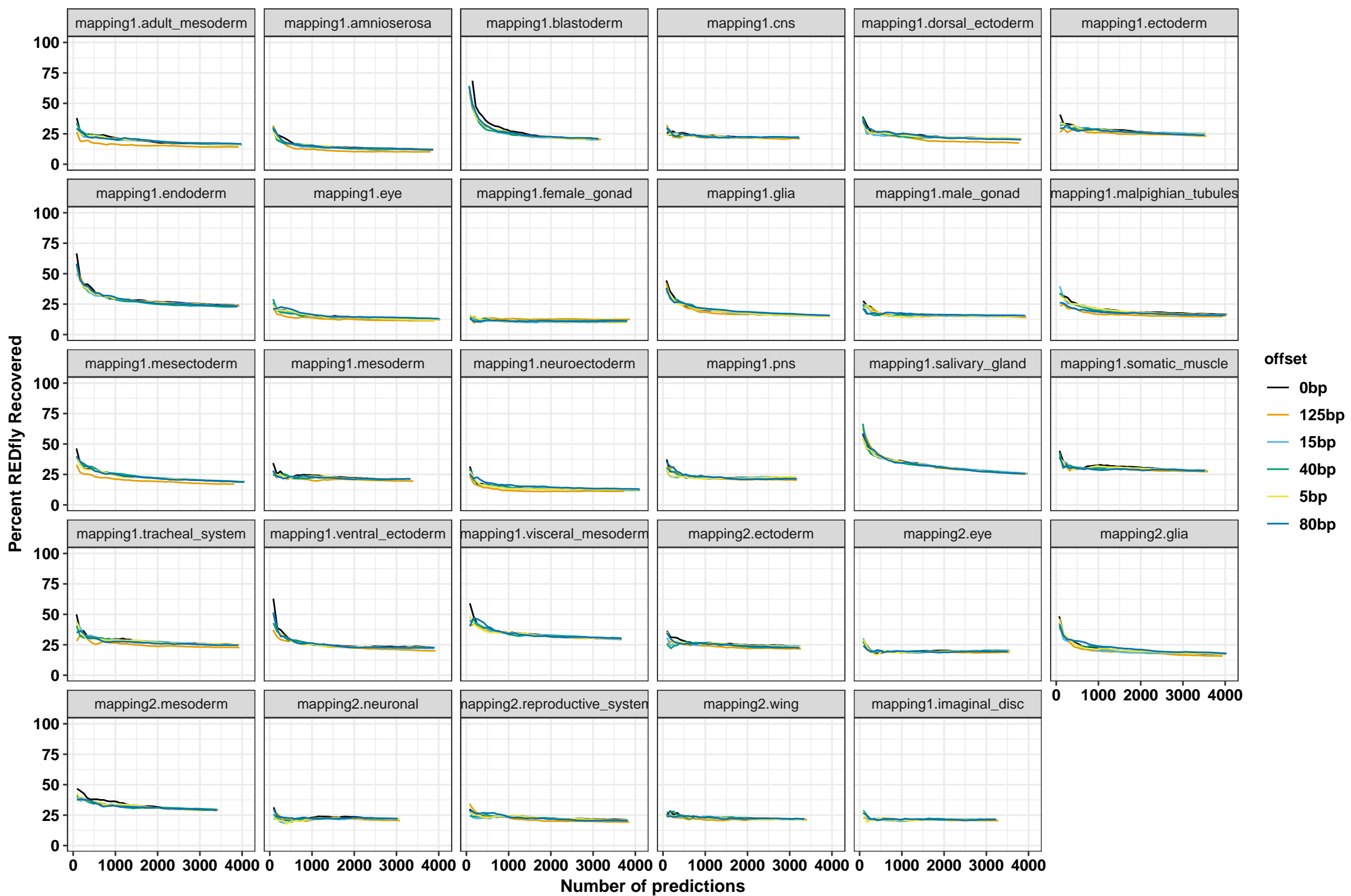

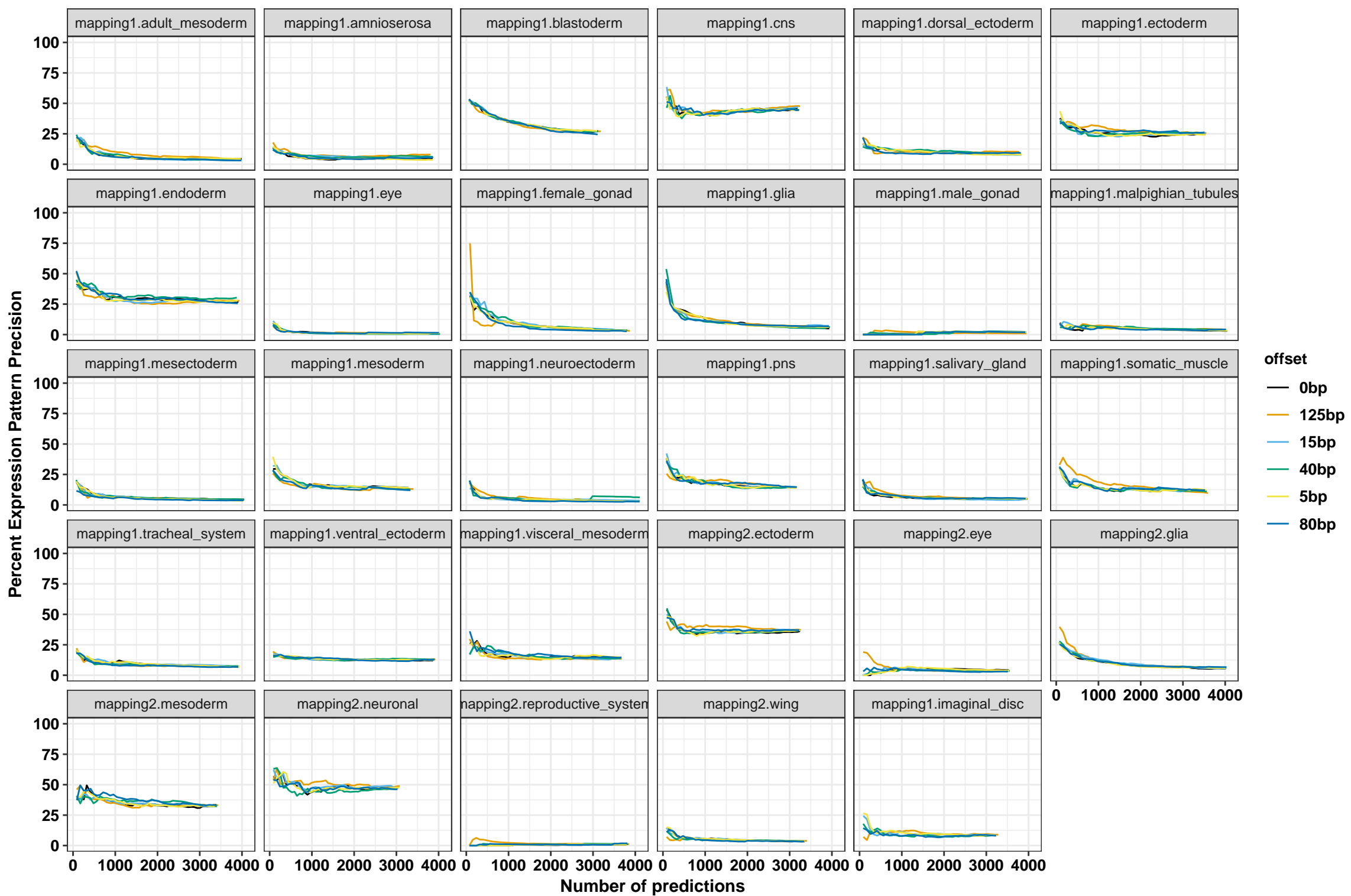

Supplement: Supplementary file 7 — Figure S3. Results of SCRMshaw assessment by pCRMeval on a semi-continuous scale. (i) Training set sensitivity, (ii) REDfly recovery, and (iii) expression pattern precision of 29 trainings sets with starting position offsets of 0, 5, 15, 40, 80 and 125 base pairs. (PDF 155 kb) [file 12859_2019_2781_MOESM7_ESM.pdf]

Figure S4, Asma and Halfon 2019

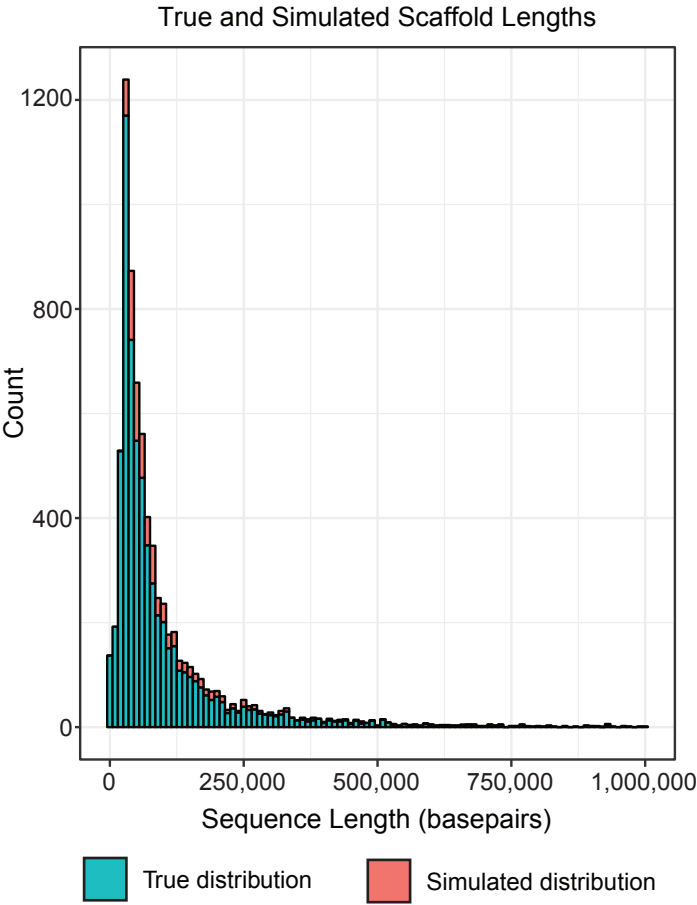

Supplement: Supplementary file 9 — Figure S4. Scaffold length distribution of real vs simulated genome. (PDF 494 kb) [file 12859_2019_2781_MOESM9_ESM.pdf]
